# Evaluating cognitive and physical work performance: A comparative study of an active and passive industrial back-support exoskeleton

**DOI:** 10.1017/wtc.2023.25

**Published:** 2023-12-20

**Authors:** Renée Govaerts, Tom Turcksin, Bram Vanderborght, Bart Roelands, Romain Meeusen, Kevin De Pauw, Sander De Bock

**Affiliations:** 1 BruBotics, Vrije Universiteit Brussel, Brussels, Belgium; 2 Human Physiology and Sports Physiotherapy Research Group, Vrije Universiteit Brussel, Brussels, Belgium; 3 Flanders Make AugmentX, Brussels, Belgium; 4 Robotics and Multibody Mechanics Research Group, Vrije Universiteit Brussel and IMEC, Brussels, Belgium

**Keywords:** Exoskeletons, device evaluation, human factors, wearable assistive device

## Abstract

Occupational back-support exoskeletons, categorized as active or passive, hold promise for mitigating work-related musculoskeletal disorders. However, their impact on combined physical and cognitive aspects of industrial work performance remains inadequately understood, especially regarding potential differences between exoskeleton categories. A randomized, counterbalanced cross-over study was conducted, comparing the active CrayX, passive Paexo Back, and a no exoskeleton condition. A 15-min dual task was used to simulate both cognitive and physical aspects of industrial work performance. Cognitive workload parameters included reaction time, accuracy, and subjective measures. Physical workload included movement duration, segmented in three phases: (1) walking to and grabbing the box, (2) picking up, carrying, and putting down the box, and (3) returning to the starting point. Comfort of both devices was also surveyed. The Paexo significantly increased movement duration in the first segment compared to NoExo (Paexo = 1.55 ± 0.19 s; NoExo = 1.32 ± 0.17 s; *p* < .01). Moreover, both the Paexo and CrayX increased movement duration for the third segment compared to NoExo (CrayX = 1.70 ± 0.27 s; Paexo = 1.74 ± 0.27 s, NoExo = 1.54 ± 0.23 s; *p* < .01). No significant impact on cognitive outcomes was observed. Movement Time 2 was not significantly affected by both exoskeletons. Results of the first movement segment suggest the Paexo may hinder trunk bending, favoring the active device for dynamic movements. Both devices may have contributed to a higher workload as the movement duration in the third segment increased compared to NoExo.

## 1. Introduction

Work-related musculoskeletal disorders (WMSDs) are a pervasive problem in today’s industries and are the primary cause of work disability, absence from work, and loss of work productivity throughout the European Union (Bevan, 2015). On average, industrial workers experience the highest prevalence of WMSDs in the back, with a 12-month prevalence of 60% (Govaerts et al., 2021). To mitigate these high numbers, companies are pursuing effective prevention strategies, such as ergonomically optimizing manufacturing workstations to limit the specific risk factors associated with developing back-related WMSDs (da Costa and Vieira, 2010). However, it is not always possible to adjust the workplace setting. Active and passive back-support exoskeletons are promising technological tools to provide assistance to the user in physically demanding tasks that cannot be sufficiently adjusted through ergonomic improvements (de Looze et al., 2016). Active back-support exoskeletons are typically powered by electrical or pneumatic sources, while passive back-support exoskeletons do not incorporate any external power sources, instead relying on deformation of springs or other elastic materials to store and release kinetic energy (de Looze et al., 2016). Both types have shown the capacity to reduce the physical load on the back (Bär et al., 2021). Yet, concentrating solely on the physical aspects falls short of depicting the comprehensive human–exoskeleton interaction. The understanding of exoskeletons’ effect on work performance plays a crucial role in determining the usability, implementation potential, and return on investment of these devices (Pesenti et al., 2021; Elprama et al., 2022).

When assessing exoskeleton effects on work performance, it is important to acknowledge that industrial tasks and work performance inherently involve both physical and cognitive components (Mehta, 2016). This blend of physical and cognitive tasks places substantial demands on workers, combining physical and cognitive workloads. While current exoskeleton research increasingly focuses on work performance (Torricelli et al., 2020; De Bock et al., 2022), cognitive demands during task simulations and performance assessments receive limited attention. Integrating both aspects would notably improve the applicability of results in real-world factory settings and provide a more comprehensive understanding of how exoskeletons impact work performance.

Furthermore, variations in support and actuation mechanisms between passive and active exoskeletons could yield distinct impacts on work-related physical and cognitive demands and therefore the overall work performance. Concerning variations in physical workload, a recent review suggested that active exoskeletons generally show greater reductions in back muscle activity compared to passive devices, although this variance may depend on exoskeleton type and task specifics (Kermavnar et al., 2021). This is further corroborated by Poliero et al. (2022), where the XoTrunk exoskeleton prototype reduced the back muscle activity almost twice as much as the passive Laevo exoskeleton (V2.56). An intuitive assumption might be that such reduction would improve the overall work performance, especially for the active exoskeleton. This is supported by other studies indicating lower energetic costs (Baltrusch et al., 2020; Schmalz et al., 2022) and delayed muscle fatigue onset (Yin et al., 2019; Lamers et al., 2020) when using a back-support exoskeleton. However, our recent study revealed that the active CrayX exoskeleton hindered overall work performance significantly more compared to the passive Paexo Back exoskeleton (Govaerts et al., 2023b). Acknowledging the contribution of participant characteristics, exoskeleton design, and experimental protocols to work performance variation, it is plausible that differences in cognitive load between the two exoskeleton types could also play a significant role. Consequently, to elucidate work performance disparities between the devices, it is imperative to further investigate this parameter.

Limited research exists regarding the disparity in cognitive demand between the two exoskeleton types. Studies on passive exoskeletons yield mixed results, depending on the specific exoskeleton and task (Madinei et al., 2020; Zhu et al., 2021; Govaerts et al., 2023a). However, Bequette et al. (2020) reported that an active lower-body exoskeleton increased cognitive demand, possibly due to its complexity, that is, the exoskeleton’s control and applied assistance, weight, bulk, and new range of motion. Active devices may generally impose higher cognitive demands compared to passive ones, as users need to interact more with the interface and learn how to adjust their movements and anticipate the exoskeleton’s responses (Stirling et al., 2019; Verl et al., 2015). Minimizing high cognitive demand is essential because exoskeletons that cause cognitive overload can lead to mental fatigue, a psychobiological state caused by prolonged periods of demanding cognitive activity (Van Cutsem et al., 2017; Habay et al., 2023). Cognitive overload and mental fatigue can negatively impact work performance by reducing attention and increasing the likelihood of errors and movement inaccuracies (Boksem et al., 2005; van der Linden and Eling, 2006). Therefore, understanding the specific aspects of work performance that are affected by exoskeletons is crucial to optimize future exoskeleton generations and ensure better compatibility with shop floors.

The objective of this study was to evaluate the impact of the net load (physical and cognitive) of active and passive back-support exoskeletons on work performance, considering both physical and cognitive aspects. We hypothesized that the CrayX would hamper both aspects of the work performance, while the passive Paexo Back would not, compared to working without exoskeleton.

## 2. Material and methods

### 2.1. Participants

Ten healthy males and six healthy females (age: 35 ± 13 years, height: 173.9 ± 8.1 cm; mass: 72.4 ± 9.5 kg) without current musculoskeletal disorders participated in this experiment. None of the participants had more than 1 hour experience with exoskeletons. Prior to participating, all participants were given information about the study and provided their consent. The experimental protocol (B.U.N. 1432022000161) was approved by the Medical Ethics Commission of the Vrije Universiteit Brussel and University Hospital Brussels.

### 2.2 Back-support exoskeletons

**Figure 1.**
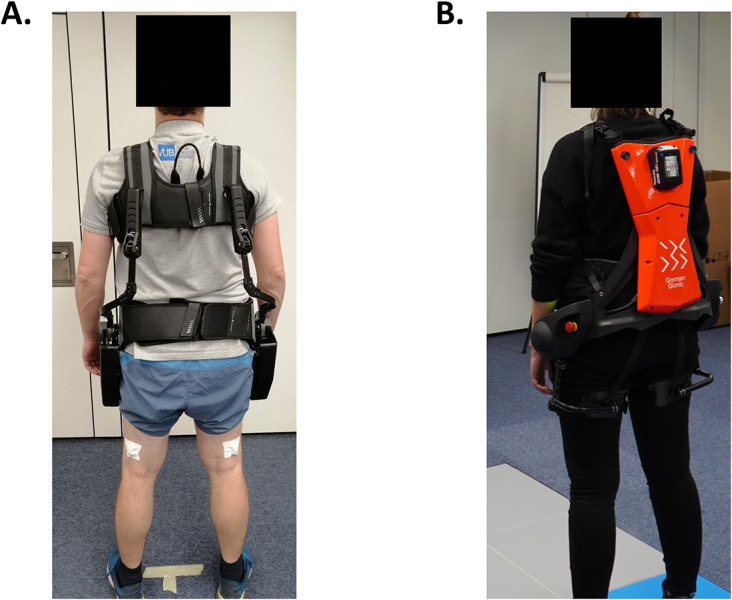
The back-support exoskeletons evaluated in this study were (a) the passive Paexo Back (Ottobock SE, Duderstadt, Germany) and (b) the active CrayX (German Bionic Systems GMBH, Augsburg, Germany).

In the present study, we employed two distinct exoskeletons (Figure 1): the passive Paexo Back (Ottobock SE, Duderstadt, Germany) and the active CrayX (4th generation, German Bionic Systems GMBH, Augsburg, Germany). A comprehensive description of these exoskeletons is provided by Govaerts et al. (2023b).

The Paexo Back (4.5 kg) provides support by creating an extension moment between the leg shells and chest vest. The level of support varies with the angle between these components. Support can be activated or deactivated using the mechanical control unit at the hip center, which can also distinguish between bending and walking, automatically turning off support during walking. In contrast to the passive spring-based design of the Paexo Back, the CrayX (7 kg) houses two electrical motors designed to align with the user’s hip joint. This active back-support exoskeleton can generate both extension and flexion moments to support the user, and the support’s magnitude remains constant throughout the movement trajectory. However, detailed information about the control system is proprietary and not publicly disclosed by the manufacturer. The Paexo Back exoskeleton was configured to operate at its maximum support level. Similarly, following consultation with the exoskeleton manufacturer, the CrayX exoskeleton was set to provide 90% support and 30% counterforce (for supporting the downward movement).

### 2.3 Experimental protocol

A randomized counterbalanced cross-over design was employed in a laboratory setting (AugmentX, Brussels). Participants completed three experimental trials, preceded by three extensive familiarization sessions to ensure sufficient familiarity with the two exoskeletons. During the familiarization sessions, participants underwent a battery of 12 functional performance tests with both exoskeletons. The 12 tasks performed can be grouped into three categories: (i) material handling (i.e., lifting, load carrying, postural tolerance tasks), (ii) occupational side activities (i.e., walking, sit to stand, stair and ladder climbing), and (iii) range of motion tasks (i.e., trunk rotation and bending, wide stance and squatting). Detailed information regarding the functional test battery can be found in Baltrusch et al. (2018). Results of the functional performance of both exoskeletons can be found in Govaerts et al. (2023b). A fourth familiarization session was conducted specifically to familiarize participants with the measurement tools and the experimental task. The subsequent three experimental conditions included (i) not wearing an exoskeleton (NoExo), (ii) wearing the CrayX exoskeleton (CrayX), and (iii) wearing the Paexo Back exoskeleton (Paexo). Rest periods of at least two days were provided between sessions to allow for adequate recovery. On the day of the experimental trials, an additional brief training session was conducted to minimize possible learning effects. This session included activities such as walking, squatting, and forward bending.

**Figure 2.**
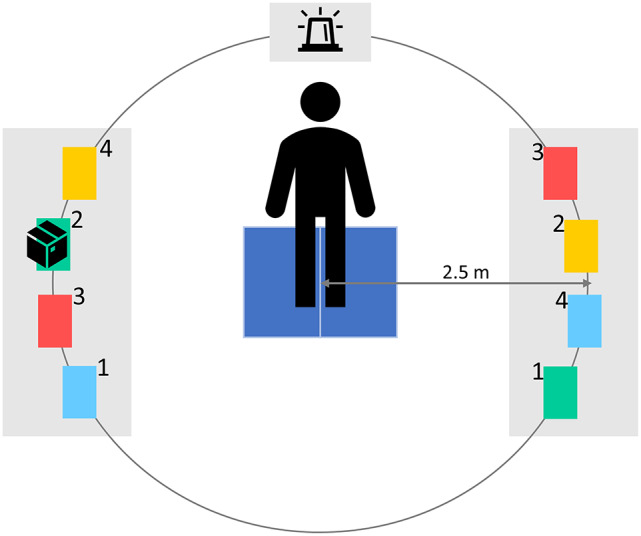
Overview experimental setup. Participants were tasked with transferring a 7 kg box in response to cues from a LED light. The LED light’s color determined the specific zone on the opposite platform to which the box needed to be transferred. The platform on the left was positioned at 14.4 cm, the one on the right at 90 cm.

Figure 2 illustrates the experimental setup. Participants were required to transfer a 7 kg box (two hand-holes L = 30 cm, W = 20 cm, H = 17 cm) between two platforms and position it on one of four delaminated zones based on the color provided by a LED light that was positioned 2.5 m in front of the participant, on a 90 cm height platform. Each trial consisted of 75 cues of the LED light delivered every 10 seconds, with each cue lasting three seconds. A photosensor was positioned on top of the LED to distinguish each cue. Four colors of the LED light were possible (randomized in order for each trial, each color was evenly present). As each of the four zones on both platforms was assigned a different color (red, blue, yellow, and green) and number (1, 2, 3, and 4), the color of the LED light indicated to which zone the box had to be transferred to:

- Red light: the box had to be transferred to the opposite platform to the zone with the same number as the current zone.
- Blue light: the box had to be transferred to the opposite platform on the zone with the same color as the current zone.
- Yellow light: the box had to be transferred to the opposite platform to the zone with an even number if the current zone of the box was uneven and to an odd number if the current zone of the box was even.
- Green light: the box had to be transferred to the opposite platform to the zone with an even number if the current zone of the box was even and to an odd number if the starting zone of the box was uneven.

**Figure 3.**
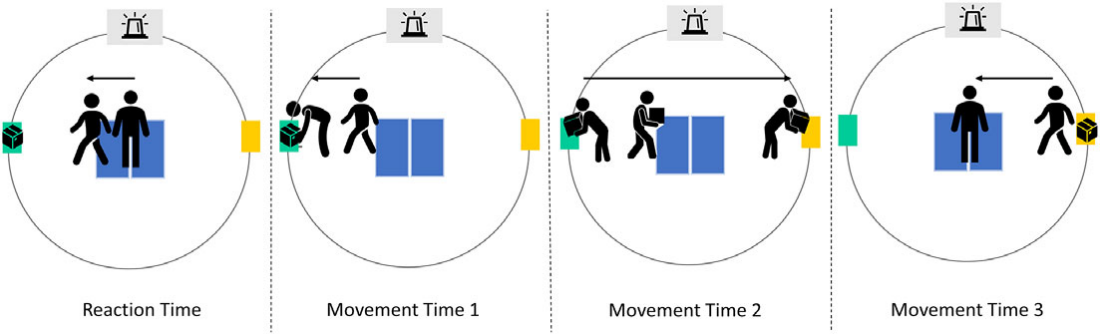
The experimental setup was simplified to improve the clarity of the transferring process. The transfer comprises four distinct movements: Reaction Time, Movement Time 1, Movement Time 2, and Movement Time 3. Reaction Time represents the duration from the activation of the LED light cue to the moment the individual steps off the force plates. Movement Time 1 denotes the interval from stepping off the force plates to lowering for box retrieval. Movement Time 2 accounts for the time taken to lift the box from one platform, transport it to the opposite platform, and place it down. Movement Time 3 signifies the duration between box placement on the platform and the individual returning to a standing position on both force plates.

The setup aimed to replicate an industrial environment and involved lifting, lowering, carrying, and walking movements (Figure 3). Two platforms were used for the experiment: an ankle-platform positioned on the left side of the participant at a height of 14.4 cm (equivalent to the height of a Euro-pallet), and a hip-platform located on the right side at a height of 90 cm. The selection of different heights was intentional, aiming to incorporate both a relatively challenging lift-and-lower movement on the ankle-platform and a simpler one on the hip-platform, where minimal lifting or lowering was required. To ensure equal walking distances to each zone, zones were positioned within a radius of 2.5 m from the fixed starting point of the participant, which was equipped with two force plates (Kistler Group 2023, Switzerland). Participants could choose their preferred lifting and lowering technique, as long as both feet maintained contact with the floor. They were instructed to respond as quickly as possible to the light cue while maintaining their preferred walking pace and ensuring that they always walked over the force plates when transferring the box. This was done to ensure a consistent walking distance for every transfer. In between movements, participants were instructed to stand in an upright position, with each foot on a force platform. The mass of the box was selected based on the NIOSH lifting equation (Waters et al., 1993).

### 2.4 Instrumentation and measurements

The movement of the box was recorded using reflective Vicon markers (diameter: 14 mm) and a Vicon system consisting of 10 large angle Vantage V5 cameras (100 Hz, Vicon, Oxford Metric Ltd., Oxford, UK). A Vicon Lock system (sampling rate: 4 kHz) was used to synchronize marker locations with force plate data and a LED light activity. The signal of the LED light was created through a voltage divider with a photosensitive element. Ground reaction forces were recorded with two 600 by 500 mm force plates (9260aa, Kistler, Switzerland). The LED light was a FitLight (FitLight training system™, Canada) LED, and its sequence was programmed using the FitLight software.

Objective outcome measurements for the cognitive component of the dual task performance included reaction time, that is, the time taken from an LED cue to stepping off the force plates, and task accuracy, that is, placing the box in the correct zone. Subjective outcome measurements, like the subjective feeling of mental fatigue, boredom, and workload, were also included. Objective outcome measurements for the physical component of the dual task performance included movement duration, that is, the moment from stepping of force plate to stepping back on it, after having transferred the box. This movement duration was further divided into three distinct components, to enhance the understanding of the impact of the exoskeletons, namely (i) Movement Time 1 (the time from stepping off the force plates to lowering to pick up the box), (ii) Movement Time 2 (the time from lifting the box from one platform, carrying it to the opposite platform, and putting it down), and (iii) Movement Time 3 (the time from when the box is put down on the platform to the moment the person is standing back on both force plates) (Figure 3). Regarding the subjective physical component, ratings of perceived exertion was chosen. Discomfort was also assessed using the body rating discomfort scale.

Regarding the subjective outcome measures, mental fatigue and boredom were assessed using visual analogue scales (VAS), which we will refer to as the Mental fatigue VAS (M-VAS) and Boredom VAS (B-VAS). Both VAS consisted of a 100 mm line with labels ranging from “not at all mentally fatigued” or “not at all boring” (score 0) to “extremely mentally fatigued” or “extremely boring” (score 100) (Smith et al., 2019). Workload was evaluated using the NASA Task Load Index (NASA-TLX), which included six subscales scored from very low (score 0) to very high (score 100) (Hart and Staveland, 1988). Rating of perceived exertion was measured using the Borg rating of perceived exertion scale (Borg-RPE), which ranged from 6 (no exertion at all) to 20 (maximal exertion) (Borg, 1982). Discomfort was assessed using the Body Part Discomfort Scale, where participants could indicate (yes/no) the body regions where they experienced discomfort when using the exoskeleton from a selection of 12 regions (Corlett and Bishop, 1976). The M-VAS was administered both before and after the dual task, while the B-VAS, NASA-TLX, Borg-RPE, and Body Part Discomfort Scale were completed after the dual task. The Body Part Discomfort Scale was only used for the two exoskeleton conditions.

### 2.5 Data analysis

Missing marker data was reconstructed using the cyclic gap filling method from the Vicon Nexus software (v2.11.0, Vicon, Oxford Metric Ltd., UK). Subsequently, marker trajectories, ground reaction forces, and cues from the LED light were exported. For all further data processing steps, custom MATLAB scripts (R20201a, The MathWorks Inc., USA) were used.

Box velocity, derived from the box trajectory, enabled the distinction between movement and stationary states by applying a 0.1 mm/s threshold. Furthermore, the signal from the photosensor placed on top of the LED light was normalized within the range [0, 1]. A 0.5 threshold was used to classify the LED as “on” (>0.5) and “off” (<0.5). Lastly, to distinguish between participants standing on and off the force plates, ground reaction force from the left and right force platforms were combined to calculate the total ground reaction force for each participant, and similar to the LED light signal, the combined ground reaction force data was normalized within the range [0, 1]. A 0.1 threshold was used to differentiate between standing on (>0.1) and off the platforms (<0.1). All created events were combined in one dataset, allowing the segmentation of each box transfer cycle into four distinct periods:

1. Reaction time: Stepping off both force plates following the LED cue.
2. Movement Time 1: Walking towards and the grasping box.
3. Movement Time 2: Picking up the box, load carrying, and putting the box down on the opposite platform.
4. Movement Time 3: Letting go of box, resuming upright position, and returning to starting position.

Task accuracy was recorded by logging box transfer errors in an Excel database, and post hoc calculations were performed to determine accuracy percentages. To capture the changes in reaction time and the three movement duration times throughout the 15-min dual task, the sequences of these parameters were divided into 3-min segments.

### 2.6 Statistics

Although we wanted to investigate changes in movement duration components over time, as all segments exhibited strong correlations (reaction time: r ≥ .72; movement duration: r ≥ .80), an overall mean score was calculated for each parameter. Normality was assessed using the Shapiro–Wilk test. If the data followed a normal distribution, a repeated measures analysis of variance (ANOVA) was conducted. For the examination of the impact of the exoskeleton condition on the reaction time variable, a one-way repeated measures ANOVA was selected. This choice was made because the platform type did not affect the reaction time since participants’ movements did not involve interaction with the platforms. A two-way repeated measures ANOVA was used to investigate (i) both the effects of exoskeleton condition and platform type on all three components of the movement duration (platform type was included since the difference in height required participants to adjust their movement according to the platform type), and (ii) the effect of time (pre and post dual task performance) and experimental condition on the M-VAS score. For both repeated measures, to explore the interaction and/or main effects, a paired sample t-test with Bonferroni corrections was conducted in a post hoc analysis.

In cases where the outcome parameter did not have a normal distribution, a Friedman test was employed. When significant, pairwise comparisons were conducted using the Wilcoxon signed-rank test with Bonferroni correction for multiple testing. Additionally, for the discomfort data, which consisted of count data, a McNemar test was used. Effect sizes were reported using partial eta squared (η^2^) or Kendall’s W (w), with the following ranges: small (η^2^ = 0.01, w < 0.3), medium (η^2^ = 0.06, w = 0.3–0.5), large (η^2^ = 0.14, w > 0.5). An alpha-error of 5% was considered as a valid cut-off for significance testing. R 4.0.3 (R Core Team, 2021) software was used to perform all data analyses and visualizations. All data are presented as means ± standard deviation.

## 3. Results

### 3.1 Reaction time

The results of the one-way repeated measures ANOVA revealed that the effect of exoskeleton condition did not reach statistical significance (F(2, 26) = 0.54, *p* = .51, η^2^ = .02), indicating that neither the passive nor the active back-support exoskeleton was associated with a significant change in reaction time and that there was no significant difference between both (NoExo = 1.35 ± 0.19 s; Paexo = 1.36 ± 0.19 s; CrayX = 1.41 ± 0.21 s).

### 3.2 Task accuracy

Task accuracy scores were positively skewed. The Friedman test showed no significant difference in task accuracy scores between the different exoskeleton conditions (NoExo = 99 ± 0.80%; Paexo = 99.1 ± 1.05%; CrayX = 99.5 ± 0.67%; X^2^(2) = 2.0, *p* = .37, w = .08).

### 3.3 Movement duration

#### 3.3.1 Movement Time 1 – Walking towards and grasping box

**Figure 4.**
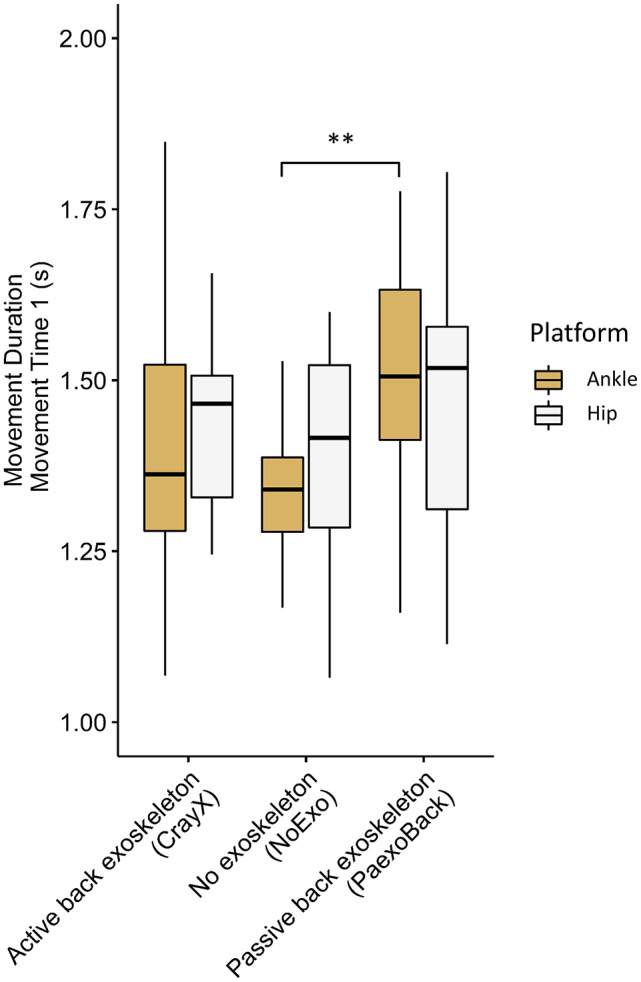
Difference in movement duration between the active back-support exoskeleton, passive back-support exoskeleton, and NoExo. Significance codes: ** (*p* < .01). The passive back-support exoskeleton hampered movement duration compared to NoExo. There was no significant effect of the active exoskeleton condition on movement duration. No significant difference between both exoskeletons was present.

A two-way repeated measures ANOVA was conducted to assess the impact of exoskeleton condition and platform height on movement duration. An interaction effect was found between exoskeleton condition and platform height (F(1.38, 16.6) = 5.67, *p* = .02, η^2^ = 0.02). Specifically, when examining the ankle-height platform, a significant difference was observed among the three exoskeleton conditions (*p* < .001). For the hip-platform, no significant difference was found (*p* = .86). Post hoc pairwise comparisons revealed that movement duration was significantly longer for the Paexo condition compared to the NoExo condition for the ankle-height platform (Paexo = 1.55 ± 0.19 s; NoExo = 1.32 ± 0.17 s; *p* < .01). Here, no other differences in movement duration were observed among the exoskeleton conditions (all *p* > .06) (Figure 4).

#### 3.3.2 Movement Time 2 – Picking up the box, load carrying, and putting the box down

The two-way repeated measures ANOVA showed no significant interaction effect between platform and exoskeleton condition with regard to movement duration (F(2, 24) = 1.45, *p* = .26, η^2^ = .002). Moreover, no significant main effects of platform (F(2, 12) = 4.73, *p* = .05, η^2^ = .004) and exoskeleton condition were present (F(2, 24) = 3.44, *p* = .05, η^2^ = .02).

#### 3.3.3 Movement Time 3 – Letting go of box and returning to starting position

**Figure 5.**
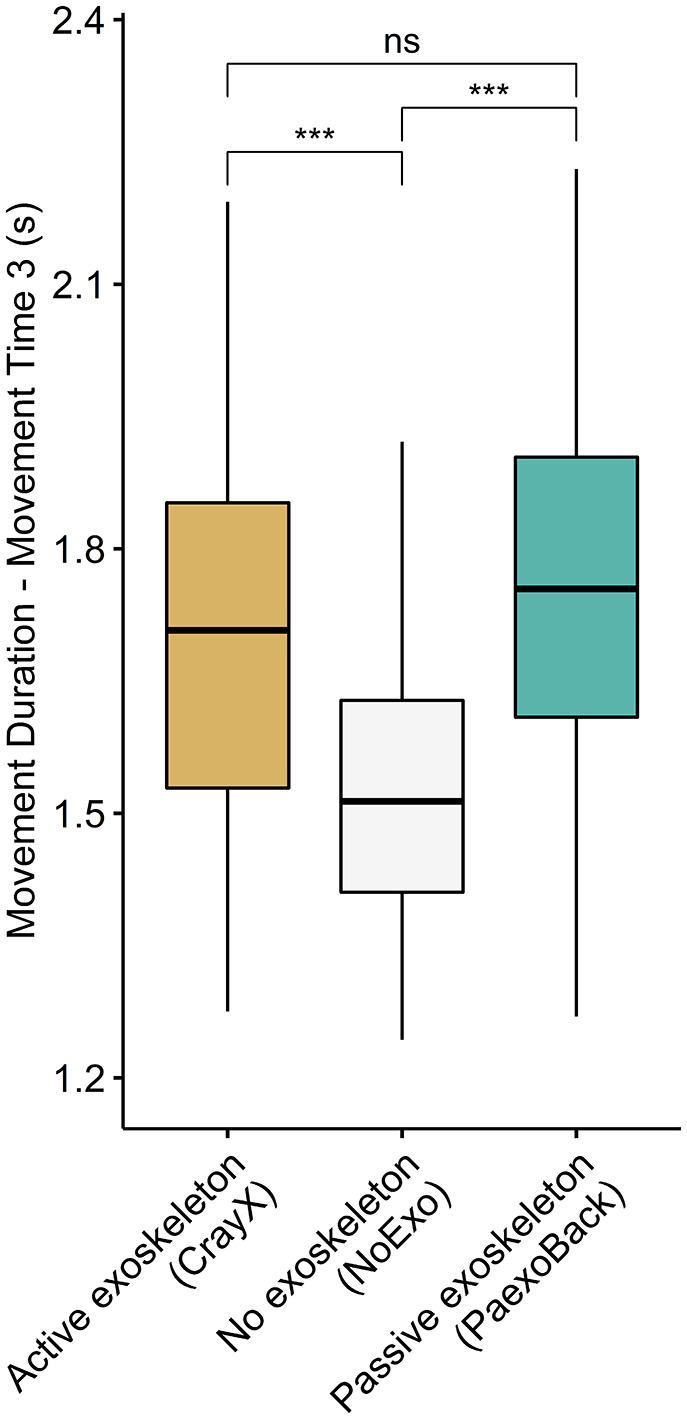
Difference in movement duration during Movement Time 3 between the active back-support exoskeleton, passive back-support exoskeleton, and NoExo. Significance codes: *** (*p* < .001). The active and passive back-support exoskeletons hampered movement duration compared to NoExo. No significant difference between both exoskeletons was present.

A two-way repeated measures ANOVA revealed no significant interaction effect between platform and exoskeleton condition with regard to movement duration (F(2, 24) = 2.18, *p* = .13, η^2^ = 0.005). Moreover, no significant main effect of platform type was found (F(2, 12) = 3.52, *p* = .08, η^2^ = 0.01). A significant main effect of exoskeleton condition was found (F(2, 24) = 8.81, *p* = .001, η^2^ = 0.12). Here, post hoc pairwise comparisons showed a significant increase in movement duration for the CrayX compared to the NoExo condition (CrayX = 1.70 ± 0.27 s; NoExo = 1.54 ± 0.23 s, *p* < .01). Additionally, a significant difference was observed between the NoExo and Paexo conditions (Paexo = 1.74 ± 0.27 s, *p* = .01). No significant difference was found between the CrayX and Paexo conditions (*p* = .98). (Figure 5).

### 3.4 Subjective measurements

#### 3.4.1 Nasa-TLX

The Friedman test showed no significant difference in Nasa-TLX scores between exoskeleton conditions with regard to all subscales: mental demand (X^2^(2) = 3.96, *p* = .14, w = .12), physical demand (X^2^(2) = 0.10, *p* = .95, w < .01), temporal demand (X^2^(2) = 0.13, *p* = .94, w < .01), frustration (X^2^(2) = 0.94, *p* = .62, w = .03), effort (X^2^(2) = 1.76, *p* = .41, w = .06), or performance (X^2^(2) = 0.49, *p* = .78, w = .02).

#### 3.4.2 M-VAS

**Figure 6.**
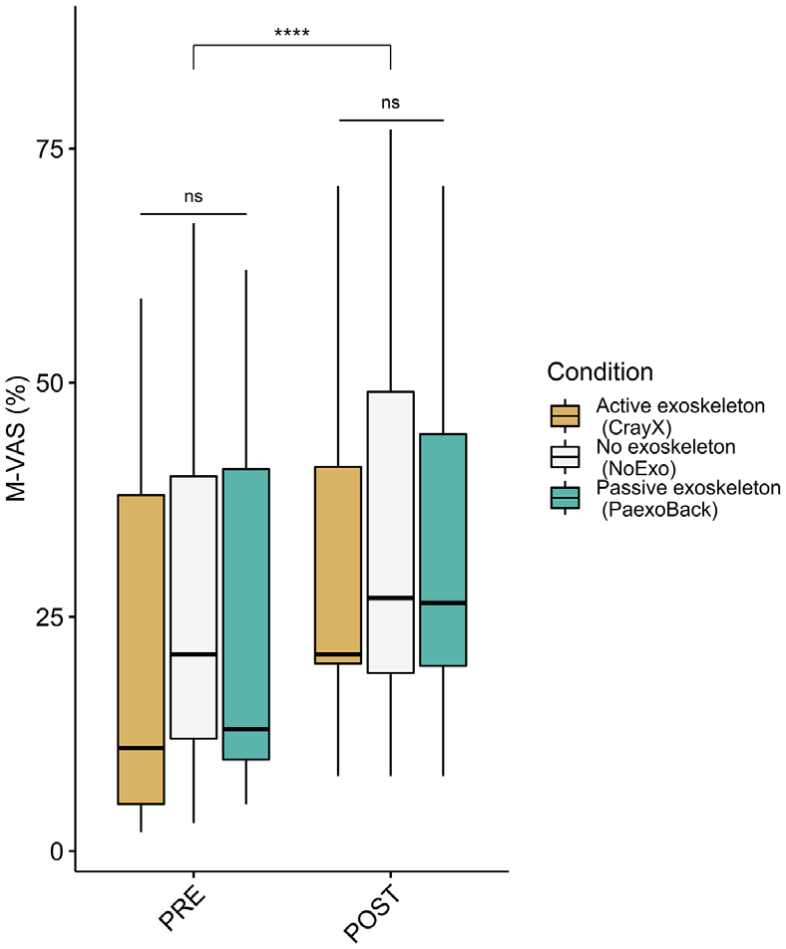
M-VAS scores according to exoskeleton condition and time-point. Significance codes: **** (*p* < .0001). No significant difference in M-VAS score was found between the active exoskeleton, passive exoskeleton, and NoExo. M-VAS scores post dual task performance significantly increased compared to pre performance. The impact of time on M-VAS scores did not vary significantly across different exoskeleton conditions.

A two-way repeated measures ANOVA revealed a significant main effect of time (F(1, 15) = 12.93, *p* < .0001, η^2^ = 0.05). There was no significant main effect of exoskeleton condition (F(2, 30) = 0.818, *p* = .45, η^2^ = 0.01). Additionally, the time:condition interaction was not statistically significant (F(2, 30) = 0.317, *p* = 0.73, η^2^ < 0.001), indicating that the effect of time on M-VAS scores did not differ significantly across the different exoskeleton conditions (Pre performance: NoExo = 27.10 ± 20.32%; Paexo = 23.13 ± 18.90%; CrayX = 20.47 ± 19.64%; *p* = .43, Post performance: NoExo = 34.71 ± 21.25%; Paexo = 32.44 ± 19.93%; CrayX = 31.12 ± 19.02%) (Figure 6).

#### 3.4.3 Discomfort

**Figure 7.**
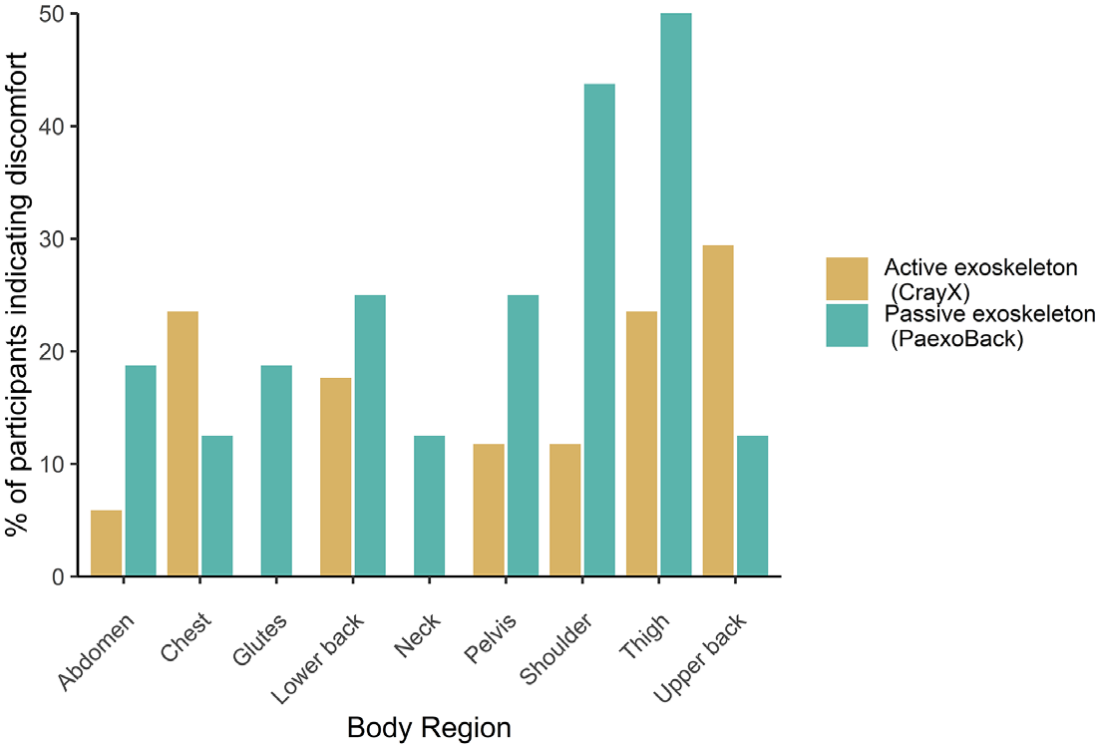
Percentage of participants indicating discomfort according to body region and exoskeleton. No significant difference between exoskeleton condition was present. Regions where no participants indicated discomfort have been omitted from the chart.

An exact McNemar’s test determined that discomfort scores did not significantly differ between the active and passive exoskeleton for all 12 body regions (all *p* > .07) (Figure 7).

#### 3.4.4 B-VAS

The results of the one-way repeated measures ANOVA revealed that boredom scores were not significantly affected by exoskeleton condition (F(2, 30) = 0.18, *p* = .84, η^2^ < 0.01).

## 4. Discussion

The aim of this study was to assess the impact of an active and passive back-support exoskeleton on both cognitive and physical aspects of work performance using a dual task. In this regard, both exoskeletons hindered the physical aspect of work performance, as evidenced by increased movement duration, without producing statistically significant results in the cognitive component of work performance compared to NoExo. Moreover, there was a notable distinction between the two exoskeletons for physical work performance outcomes. Specifically, for Movement Time 1, the passive Paexo Back exoskeleton increased movement duration in comparison to NoExo, whereas the CrayX exoskeleton retained from significantly impacting movement duration. Movement Time 2 was not significantly different across experimental conditions, while Movement Time 3 was significantly longer for both exoskeletons compared to NoExo, with no significant differences between the exoskeletons. Lastly, no significant differences were observed between the CrayX, Paexo Back, and NoExo in terms of subjective measurements.

### 4.1 Movement Time 1 - Walking towards box and grasping box

The observed increase in movement duration with the Paexo Back appears to be primarily linked to its spring-based design. Although further motion analysis is needed to corroborate this hypothesis, it is known that the specific design introduces resistance during hip and trunk flexion, which could be noticeably experienced when grasping the box on the ankle-height platform. Unlike previous generation exoskeletons, for example, the Laevo (V2.0 and V2.5, Laevo B.V., Delft, Netherlands) and BackX (Ottobock SE, Duderstadt, Germany), two passive back-support exoskeletons known to impede walking performance (Baltrusch et al., 2018; Luger et al., 2021; Park et al., 2022), the Paexo Back incorporates a mechanical clutch at the hip that automatically distinguishes between bending and walking and switches itself off during walking. This addition seems to effectively mitigate hindrances to walking, as previously indicated in our earlier study (Govaerts et al., 2023b).

Furthermore, the issue of passive devices providing unwanted resistance to dynamic movements has been discussed by other researchers, therefore favoring these exoskeletons for static tasks (Tommaso Poliero et al., 2022; Toxiri et al., 2019). However, the Paexo Back demonstrated an overall positive trend in work performance and perceived task difficulty during repetitive lifting and lowering of a 20 kg box in our previous study (Govaerts et al., 2023b). Furthermore, in similar studies investigating passive devices (including the Paexo Back) for comparable lifting and lowering tasks, significant reductions in muscle activity and energetic expenditure were observed (Alemi et al., 2020; Luger et al., 2021). These findings suggest that the benefits of the Paexo Back could extend beyond static tasks as further corroborated by the absence of a significant difference in movement duration for the two different platforms in Movement Time 2. This observation implies that when executing the lowering movement with a certain weight, the resistance constraints of the device are reduced. In fact, the device could potentially provide support to the back muscles engaged in eccentric contractions. Yet, achieving a balance between dynamic task support and mitigating hindrance remains complex. This challenge is evident in other passive exoskeletons, which, while reducing muscle activity, often led to extended movement durations in diverse dynamic tasks (Baltrusch et al., 2018; Luger et al., 2021). Efforts to alleviate this challenge involve adjustable support levels, albeit within constrained choices. However, tasks that encompass multiple subtasks like lifting, lowering, walking, and load carrying seldom allow real-time support adjustments (Crea et al., 2021). In such scenarios, active devices with intelligent actuation capabilities are likely to exhibit greater potential (Toxiri et al., 2019; Crea et al., 2021).

The active CrayX exoskeleton did not significantly increase the forward bending or walking movement; Movement Time 1 exhibited no noteworthy change compared to the NoExo condition. Although no hindrance during squatting or forward bending was anticipated, walking performance outcomes contrast with our earlier findings, which reported a significant impediment due to the CrayX exoskeleton (Govaerts et al., 2023b). While hypothesized to introduce a certain level of resistance to walking, a challenge commonly faced by other active devices such as the XoTrunk (the latter decreased stride speed due to a corresponding increase in stride duration, ranging between 6% and 8% (Poliero et al., 2020)), it is plausible that the relatively short segmented walking distance (2.5 m) might have constrained the potential for significant differences. Alternatively, the task goal in this case might have been more demanding compared to the sole act of walking over a distance, as investigated by Govaerts et al. (2023b) and Poliero et al. (2020). This could have motivated participants more, thereby improving their performance scores (Locke et al., 1981). However, it is also possible that we should further interpret this finding in accordance with Movement Time 2 and 3.

### 4.2 Movement Time 2 and 3

To link the lack of significant results in Reaction Time, task accuracy and Movement Time 2 with the increased movement duration in Movement Time 3 for both exoskeletons, independent of platform height, the possibility of both exoskeleton increasing cognitive and/or physical workload should be considered. To cope with this additional workload while still meeting task requirements, participants could have adjusted their performance strategy. By doing so, they would have avoided excessive exertion, thus preserving their effort (Geurts and Sonnentag, 2006; Meijman and Mulder, 2013). Here, this was translated in an increased Movement Time 3, that is, after placing the box on the opposite platform, hereby completing the most important part of the task, participants walked slower back to the starting point. This slower task pace is a common coping strategy (Geurts and Sonnentag, 2006). However, due to ambiguity of work performance effects, it becomes more and more clear that the user, the exoskeleton, and even the types of tasks are all contributing factors in a possible work overload. This again highlights the importance of considering each exoskeleton implementation case by case. Moreover, designers should be encouraged to limit additional physical load (e.g., by avoiding resistance to the walking movement), and cognitive load (e.g., through an intuitive user interface) when designing exoskeletons. Most importantly, one should consider the adaptation period of users to get acquainted with the device as the users’ perception of workload may change over time when becoming more proficient with the exoskeleton. Familiarization periods varies strongly in literature, hereby contributing to the ambiguity in results. Therefore, standardization of human–exoskeleton familiarization levels (Moyon et al., 2019), should be pursued.

### 4.3 Difference between passive Paexo Back and active CrayX

Although both exoskeletons increased workload, the possibility of variations in the additional workload within the two categories persists. In this specific task, we hypothesize that fitting challenges and increased discomfort associated with the passive device could have led to a higher cognitive demand compared to the CrayX. Previous research (Baltrusch et al., 2018; Huysamen et al., 2018; Govaerts et al., 2023b) similarly underlined issues concerning the fitting of passive back-support exoskeletons, especially regarding the placement of the hip belt, which could potentially shift upward. In our study, we encountered similar issues, occasionally leading to misalignment between the exoskeleton joints and the corresponding human joint. This misalignment led to audible noise during walking and bending, potentially demanding more attention from participants compared to the CrayX. Moreover, this could have also provided some level of additional resistance. The CrayX, on the other hand, did not encounter fitting challenges, and because participants were not required to engage with the device (e.g., adjust the fitting or change support levels), it likely demanded less attention, hereby minimizing the cognitive load. Furthermore, considering that pain tends to capture attention (Eccleston and Crombez, 1999), the observable trend of more participants expressing discomfort with the Paexo Back than with the CrayX suggests that participants may have focused more on adjusting the Paexo Back for enhanced fit and decreased discomfort (Stirling et al., 2020). Conversely, we also posit that the physical load, stemming from the larger mass and more restricted range of motion imposed by the CrayX (Govaerts et al., 2023b), might have resulted in higher physical demand on users compared to the Paexo. Consequently, these factors might have eventually counterbalanced the disparities in work performance between the two devices.

### 4.4 Limitations and future research

Though the sample size is moderately limited, it aligns with the scope of previous ergonomics studies focusing on human–exoskeleton interaction. These outcomes unveiled notable diversity, as highlighted by the substantial standard deviations, particularly in movement duration and M-VAS scores. This indicates that people exhibit distinct reactions to exoskeletons. Nevertheless, the sample size employed in this study did not permit a comprehensive exploration of possible underlying elements that could account for these individual disparities. Moreover, even though we implemented a relatively thorough familiarization process compared to other evaluation studies, results still reveal immediate effects of the exoskeleton interaction. The lack of available data on the optimal duration for exoskeleton familiarization presents a difficulty in establishing the ideal familiarization period. This predicament is compounded by substantial variations in reported durations across different studies (Baltrusch et al., 2018; Madinei et al., 2020; Bär et al., 2021; Luger et al., 2021; Park et al., 2022; Schmalz et al., 2022). These disparities, coupled with other divergences in study designs, contribute to the complexity of comparing results across exoskeleton studies. Hence, it becomes essential to conduct investigations into the learning curve necessary for individuals to proficiently engage with exoskeletons. Moreover, long-term studies to evaluate the sustained implications of exoskeleton implementation should be performed to understand differentiate between acute and chronic effects of exoskeleton on work performance. While the results of this experiment offer valuable insights into the cognitive and physical dimensions of work performance and the influence of exoskeletons, it is important to acknowledge that outcome parameters could have been impacted differently with an increased task complexity (task accuracy scores consistently exceeded 99%). Here, expanding the comprehension of how workload was influenced by these devices would be advantageous. This objective could be further pursued by incorporating supplementary objective measurements, such as including muscle activity through electromyography and monitoring brain activity via an electroencephalogram or functional near infrared spectroscopy. Moreover, further motion capture analysis in order to break down the movement cycle into more detail (i.e., lifting, lowering, walking, and load carrying) could provide more insights regarding the suggested hypotheses.

## 5. Conclusion

The present study found no significant difference in cognitive work performance, during the execution of a simulated material handling task, between the passive Paexo Back, active CrayX exoskeletons, and NoExo. Moreover, movement duration for the majority of the movement cycle, that is stepping of the force plates to transferring the box to the opposite platform, did not significantly differ between exoskeleton conditions. However, after transferring the box, when participants had to walk back to the starting point (= Movement Time 3), movement duration significantly increased for both exoskeletons compared to NoExo. This suggests that participants likely experienced an additional load, which could be physical and/or cognitive. To maintain their performance level throughout the 15-min dual task, participants seemingly used Movement Time 3 as a recovery period. These findings hold important implications for companies and users, especially when sufficient recovery is not available, as it could potentially lead to hampered work performance when implementing exoskeletons. Furthermore, the present study demonstrates a significant difference between the passive Paexo Back and active CrayX exoskeleton in terms of the physical aspect of work performance, more specifically the resistance to the trunk bending movement, favoring the flexibility of the active device’s actuation control over the spring-based actuation system of the passive device. For working tasks involving predominantly dynamic movements with little range of motion requirements, for example, symmetrical lifting/lowering, active back-support exoskeletons, such as the CrayX, can really be considered. The complexity of this topic stresses the importance of a structured implementation process, where employees are well-informed, the work situation is well-studied, and the staged implementation process is closely monitored.

## Data Availability

Raw data were generated at Flanders Make AugmentX Research lab. Derived data supporting the findings of this study are available from the corresponding author K.D.P. on request.
